# Dysregulation of Metabolic Peptides in the Gut–Brain Axis Promotes Hyperinsulinemia, Obesity, and Neurodegeneration

**DOI:** 10.3390/biomedicines13010132

**Published:** 2025-01-08

**Authors:** Camille Green, Vandana Zaman, Kayce Blumenstock, Narendra L. Banik, Azizul Haque

**Affiliations:** 1 Department of Neurosurgery, Medical University of South Carolina, 96 Jonathan Lucas Street, Charleston, SC 29425, USA; greecami@musc.edu (C.G.); zamanv@musc.edu (V.Z.); baniknl@musc.edu (N.L.B.); 2 Ralph H. Johnson Veterans Administration Medical Center, 109 Bee Street, Charleston, SC 29401, USA; kab319@musc.edu; 3 Department of Pharmacology and Immunology, Medical University of South Carolina, 173 Ashley Avenue, Charleston, SC 29425, USA

**Keywords:** hormonal peptides, hyperinsulinemia, diabetes, inflammation, gut–brain axis, neurodegeneration

## Abstract

Metabolic peptides can influence metabolic processes and contribute to both inflammatory and/or anti-inflammatory responses. Studies have shown that there are thousands of metabolic peptides, made up of short chains of amino acids, that the human body produces. These peptides are crucial for regulating many different processes like metabolism and cell signaling, as they bind to receptors on various cells. This review will cover the role of three specific metabolic peptides and their roles in hyperinsulinemia, diabetes, inflammation, and neurodegeneration, as well as their roles in type 3 diabetes and dementia. The metabolic peptides glucagon-like peptide 1 (GLP-1), gastric inhibitor polypeptide (GIP), and pancreatic peptide (PP) will be discussed, as dysregulation within their processes can lead to the development of various inflammatory and neurodegenerative diseases. Research has been able to closely investigate the connections between these metabolic peptides and their links to the gut–brain axis, highlighting changes made in the gut that can lead to dysfunction in processes in the brain, as well as changes made in the brain that can lead to dysregulation in the gut. The role of metabolic peptides in the development and potentially reversal of diseases such as obesity, hyperinsulinemia, and type 2 diabetes will also be discussed. Furthermore, we review the potential links between these conditions and neuroinflammation and the development of neurodegenerative diseases like dementia, specifically Parkinson’s disease and Alzheimer’s disease.

## 1. Introduction

Metabolic peptides have become a novel research area for the potential treatment of metabolic diseases such as hyperinsulinemia, diabetes, obesity, and dementia. Glucagon-like peptide-1 (GLP-1) is a pleiotropic hormone that is well known for its incretin effect in the glucose-dependent stimulation of insulin production [1,2,3]. Additionally, GLP-1 is also produced in the brain and plays an essential role in neuroprotection and inflammation through its activation of GLP-1 receptor signaling pathways. It is a versatile hormone with broad pharmacological potential and displays several metabolic effects. A number of in vivo and in vitro studies using preclinical models of inflammatory and neurodegenerative diseases show that GLP-1 receptor (GLP-1R) activation has anti-inflammatory properties [3,4,5]. L cells produce GLP-1, which binds to the GLP-1R found on afferent neurons within the intestine and portal vein. L cells are cells in the gut that produce hormones that regulate metabolism and physiology [6]. While L cells are mainly found in the large intestine and ileum, they can also be found in the duodenum and jejunum. The thymus, spleen, and different immune cells, including monocytes, macrophages, natural killer T cells, and T cells, all contain GLP-1R. GLP-1 can also act as a key regulator of the gut–brain axis and may influence the brain indirectly through vagal nerve fibers in the enteric area [7,8]. Since GLP-1 is quickly metabolized and rendered inactive by dipeptidyl peptidase IV upon release into the extracellular space, its short half-life restricts its potential for therapeutic use. In recent years, several pharmacological agents, including GLP-1 derivatives, have been developed to treat obesity and type 2 diabetes mellitus (T2DM) with improved bioavailability [9,10]. The activation of GLP-1R is also known to induce cellular responses, leading to neuroprotection, neurodevelopment, memory formation, cell growth, synapse growth, repair, and regeneration. It also increases the release of neurotransmitters and activates Ca^2+^ channels. Thus, GLP-1 emerges as a therapeutic target for treating inflammatory diseases in the gut and brain.

Gastric Inhibitory Polypeptide (GIP) is a hormone released by cells in the gut that helps regulate blood glucose levels and nutrient balance [11,12]. It is believed to have positive effects on lipid metabolism, bone strength, cardiovascular function, and cognition. GIP is also an incretin hormone released by enteroendocrine K cells in response to feeding and can stimulate insulin release to regulate blood glucose and nutrient homeostasis [11,13]. This is thought to represent indirect effects (mediated by reductions in insulin resistance) and possible direct effects (via surgically induced changes in GIP, GLP-1, and affiliated peptide hormones) on the ovaries and the hypothalamic–pituitary–adrenal axis. Despite the known physiological role of GIP as an insulin secretagogue that controls postprandial blood glucose levels, emerging evidence reveals important actions of GIP in relation to adipocytes and the upregulation of fat deposition in tissues [14]. Thus, blocking GIP receptor function could be used as a tool to counter insulin resistance and improve metabolic status in obesity and related diabetes. GIP can be secreted into the circulation in response to the ingestion of glucose or fats, and acts in concert with GLP-1 to induce the incretin effect by amplifying glucose-stimulated insulin secretion [15,16]. This effect is now recognized to be more physiologically significant than GIP’s acid-inhibiting effects, and is related to glucose-dependent insulinotropic polypeptides.

Pancreatic polypeptide (PP) is an endogenous hormonal peptide released by F cells [17,18]. F cells are cells in the pancreatic islets (islets of Langerhans) of the pancreas. They help synthesize and regulate the release of PP hormones. The Y4 receptor, which is expressed in the liver, intestine, adrenal gland, and certain areas of the CNS, can bind to PP. It is important to mention that PP plays an important role in regulating glucose. By increasing the number of insulin receptors in the hepatocytes, the peptide makes the liver more sensitive to the effects of insulin. PP also has a variety of other actions in vivo, including the inhibition of pancreatic secretion, the inhibition of gallbladder contraction, the inhibition of gastric motility, and the inhibition of acid secretion [19]. Since the administration of PP does not elevate serum insulin levels, PP therapy is regarded as safe [17]. PP has immense potential with multiple mechanisms of action in the treatment of pancreatogenic diabetes, which is difficult to diagnose and manage. The injection of PP into wild and genetically obese mice revealed long-lasting effects of reduced food intake, decreased weight, and improved glucose and lipid profile [20]. Metabolic peptides such as GLP-1, GIP, and PP play crucial roles in regulating body weight, glucose homeostasis, obesity, and inflammation.

Obesity affects more than 40% of people in the US, and it can lower life expectancy by 5–10 years. Peptide drugs have recently grown as a field in the treatment of obesity, as pharmacological modulators of food intake and regulators of body weight. Obesity is a chronic relapsing, multi-factorial, neurobehavioral disease that forms as the result of excessive fat accumulation in the body [21,22,23]. This disease affects 1 in 5 children and 2 in 5 adults and is usually assessed by measuring one’s body mass index and height [24,25]. Some common factors that have been discovered to lead to the development of obesity include the overconsumption of calories through things like fast food and other processed foods, the overconsumption of sugary foods and drinks, hormonal changes, and certain medications [23,26]. There are even psychological factors, such as boredom, loneliness, anxiety, depression, and more, that can play a part in the buildup of an obesity diagnosis [23,26]. Obesity has been discovered to have detrimental effects on various body functions, increasing one’s risk of premature death; having obesity can cause the body to develop conditions like asthma, osteoarthritis, and sleep apnea, since excess body fat can overpopulate around the organs of the respiratory system, and this can also cause unnecessary pressure on the musculoskeletal system [26,27,28]. Obesity can also cause metabolic changes that allow for the development of cardiovascular disease, heart failure, heart attacks, stroke, kidney disease, and many more. Two more important risks to acknowledge regarding the severity of obesity include the potential development of T2DM and/or chronic inflammation revolving around hyperinsulinemia. There has been crucial research undertaken on these conditions that has allowed researchers to theorize that there may be a link between T2DM and neuroinflammation that can lead to diseases like dementia, specifically Parkinson’s disease (PD) and Alzheimer’s disease (AD) [29,30,31]. Research has also led to a better understanding of the roles that distinct metabolic peptides have, regarding specific conditions like inflammation and diseases.

## 2. Hyperinsulinemia, Inflammation, and Diabetes

The National Institute of Diabetes and Digestive and Kidney Diseases (NIDDK) states that T2DM is a condition in which the pancreas does not make enough insulin to help glucose reach cells in the body, and this causes the blood glucose or blood sugar level to be extremely high [32,33]. Research performed on hyperinsulinemia and T2DM has concluded that they are both conditions which pertain to insulin regulation—or the lack thereof [34,35]. Even though hyperinsulinemia is usually associated with T2DM, different processes take place for each respective condition. In hyperinsulinemia, the body does not respond properly to the production of insulin from the pancreas, and it develops insulin resistance [36]. Due to this, the pancreas tries to overcompensate by producing even more insulin in an attempt to break the resistance, but this just leads to higher insulin levels in the blood, and this can put a person at risk of T2DM.

Researchers have been working on recognizing patterns that occur in specific cases of obesity leading to hyperinsulinemia and or T2DM, and then connecting this to inflammatory diseases, specifically neuroinflammatory diseases, highlighting the gut–brain axis [34,37]. By understanding the gut–brain axis more clearly, researchers will potentially be able to link conditions like obesity and diabetes to neuroinflammatory diseases such as AD. Once this happens, they will likely use knowledge of the roles metabolic peptides play and link this to reversing the effects of conditions like diabetes, thus producing groundbreaking research that will possibly result in the prevention of the development or progression of certain neuroinflammatory diseases. Metabolic peptides are often implicated in the dysregulation of signals in the gut–brain axis and inflammatory diseases such as cardiovascular disease and AD. Regarding inflammatory diseases, researchers have been working to further understand the role of specific metabolic peptides such as GLP-1, GIP, and PP [34,37]. This review article will focus on the specific functions of GLP-1, GIP, and PP metabolic hormones, and their implications in hyperinsulinemia, obesity, and dementia.

## 3. GLP-1 in Hyperinsulinemia and Diabetes

**Figure 1:**
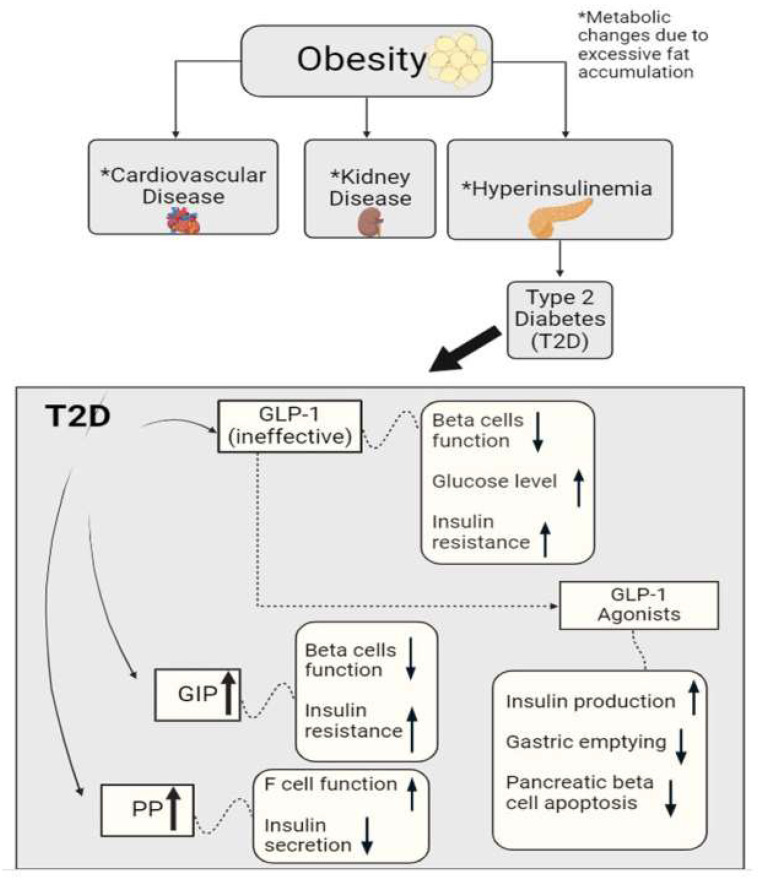
Graphical representation of metabolic hormonal peptides glucagon-like peptide 1 (GLP-1), gastric inhibitor polypeptide (GIP), and pancreatic peptide (PP), and their roles in metabolic homeostasis. Alterations in these peptides may trigger cardiovascular disease, kidney disease, hyperinsulinemia, etc. These metabolic hormones can also regulate F-cell and β-cell functions and insulin sensitivity/resistance in diabetes.

GLP-1 is a peptide hormone that normally stimulates the secretion of insulin, as well as suppresses glucagon production and delays gastric emptying, resulting in a decreased amount of food intake [38]. A study performed on the secretion of GLP-1 in patients with T2DM found that the patients generally did not have a reduced secretion of GLP-1 in response to oral glucose or liquid meal tests; however, they had significantly low glycemic control responses, meaning that the effectiveness of the GLP-1 that was being secreted was definitely compromised [39]. These findings help researchers further understand GLP-1 and the role that its receptors and agonists play in conditions like T2DM, as shown in Figure 1. GLP-1 agonists, more commonly known as drugs to treat diabetes, mimic the activity of GLP-1 and bind to receptors in an attempt to lower glucose levels and manage metabolism in patients with T2DM [40]. GLP-1 agonists not only stimulate insulin secretion and slow gastric emptying, but also decrease pancreatic beta (β) cell apoptosis, which is significant since pancreatic β cell function is needed to efficiently manage and control blood glucose levels [41,42]. Researchers have been able to see the benefits of GLP-1 agonists reach further than just improving insulin production and promoting weight loss; they have seen them potentially decrease the risks of different inflammatory diseases, including those that revolve around neuroinflammation [43,44].

## 4. GIP in Hyperinsulinemia and Diabetes

GIP is an incretin hormone that is involved in the regulation of lipid metabolism and the facilitation of fat deposition in the body [45,46]. This hormone plays a huge role in stimulating glucose-dependent insulin secretion as it induces β cell proliferation and inhibits apoptosis as well. In a study performed on whether the inhibition of GIP signaling prevents obesity, researchers found that an excessive accumulation of fat allows for the hypersecretion of GIP [47,48]. This hypersecretion results in an increase in nutrient uptake in cells specialized in fat storage (adipocytes), thus becoming a major factor in the development of obesity, as researchers have found that obese patients have a higher level of GIP [47]. The insulin resistance that takes places here leads to hyperinsulinemia and potentially diabetes, further inducing an increase in nutrient uptake into adipocytes. Researchers have found that in subjects with GIP receptor deficiency, the fat in their bodies is not facilitated efficiently, resulting in higher adiposity. In patients with T2DM, there is resistance to the effect of GIP in the body (Figure 1). As the body tries to recompensate and produce more GIP, β cells’ functioning plummets and insulin resistance rises.

However, like with GLP-1, researchers have been able to create GIP receptor agonists to combat the body’s resistance to GIP, but more extensive research must be undertaken, since an increased amount of GIP can cause even more issues with insulin resistance and fat deposition [45,49]. It is also noted that any use of GIP agonists should be used paired with GLP-1 agonists for the most efficient treatment, enhanced by their combined insulinotropic effects. On the other hand, the creation of GIP antagonists has provided great results regarding the understanding of combatting T2DM. GIP antagonists will bind to GIP receptors, blocking the present GIP agonists from further activity [50]. This process is very important for patients with T2DM, as this allows for GIP levels to decrease, which will lower insulin resistance and blood glucose levels, increase the productivity of GLP-1, improve β cell function, and even decrease body weight [48,49,50,51]. Along with further understanding its insulinotropic factors, researchers have also been able to discover the role that GIP plays in neuroinflammation, as it has been revealed to potentially exhibit neuroprotective and pro-inflammatory properties [3,52,53].

## 5. PP in Hyperinsulinemia and Diabetes

PP is a peptide hormone that is rapidly secreted from the endocrine pancreas after a meal has been ingested [54,55,56]. It has been discovered that its roles consist of regulating intestinal motility, metabolism, and delaying gastric emptying, therefore decreasing food intake. As a response to elevated blood glucose levels, there is an increase in the function of cells in the pancreatic islets of the pancreas (F cells), stimulating the release of PP [57]. When a patient has hyperinsulinemia or T2DM, it is found that PP is secreted more abundantly from the pancreas due to the increased amount of insulin because of insulin resistance and high levels of blood glucose [57,58]. This creates a cycle in the patient, as an elevated level of PP causes insulin secretion to be diminished and it also increases the functionality of F cells, which allows for the production of even more PP (Figure 1). Chronic pancreatitis has been linked with lowered plasma levels and a blunted nutrient-induced release of PP. Studies have shown that chronic pancreatitis accompanied by PP deficiency could be associated with partial hepatic resistance both in the basal state and in response to hyperinsulinemia [59,60,61]. This impairment was reversed after intravenous PP administration, suggesting that PP deficiency may play a role in the development of pancreatogenic diabetes caused by pancreatic injury.

A recent study tested the effect of extended PP administration in patients whose entire insulin requirement was exogenous and, therefore, was deemed measurable [62]. This study showed that a continuous subcutaneous infusion of PP increased insulin sensitivity and resulted in a reduction in daily insulin requirements in a varied group of T2DM and type 3c diabetes mellitus (T3cDM) patients on insulin pump therapy. Thus, the effectiveness of PP infusion in decreasing insulin requirements could be correlated with the degree of PP deficiency present in these patients, suggesting a possible therapeutic role of PP in type 1 diabetes mellitus (T1DM), as well as T3cDM patients.

## 6. Metabolic Peptides in Inflammation and Neurodegeneration

When the central nervous system (CNS) has been damaged or is encapsulated by inflammation, the brain develops gliosis as a defensive/protective immune response [63]. Gliosis is known as a reaction that attempts to combat the degeneration and necrosis of cells in the CNS by increasing the proliferation of large glial cells, as it aims to restore homeostasis within the cells and limit/prevent further tissue damage [64,65]. However, due to its fibrous nature and large size, gliosis produces scarring formed by astrocytes and, depending on the extensiveness of the damage, this scarring can have negative effects on various processes in the CNS [66]. When damage to the CNS is not treated in a specific amount of time, gliosis has been found to limit synaptic regeneration after injury, limit axonal regeneration, limit regeneration and functional recovery after spinal cord trauma, and restrict the integration of neural grafts and neural stem/progenitor cells [67]. These factors raise the risk of neuroinflammation and neurodegeneration significantly.

**Figure 2:**
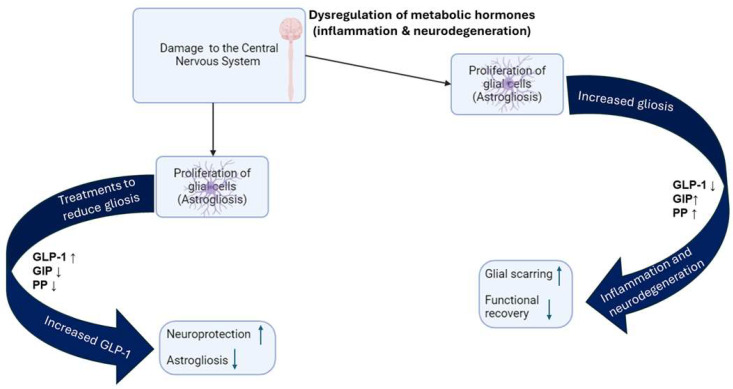
Dysregulation of glucagon-like peptide 1 (GLP-1), gastric inhibitor polypeptide (GIP), and pancreatic peptide (PP) disrupts cellular functions and contributes to the development of various inflammatory and neurodegenerative diseases.

Our recent study on the effects of enolase inhibition (ENOblock) on metabolic hormones and inflammatory factors after spinal cord injury in rat models found that the treatment of ENOblock enforced neuroprotection and hindered the effects of astrogliosis by means of slowing the formation of glial scarring [68]. We were able to make a connection between the decrease in gliosis and the increased levels of metabolic peptides like GLP-1, whose receptors are found on microglia and astrocytes, as well as GIP (found on neuronal cells), which has also been proven to reduce astrogliosis [68,69,70,71]. We were also able to see a decrease in GLP-1 in the injured rats, but an increase in GLP-1 in the ENOblock-treated rats, resulting in significant neuroprotectant properties, especially within neuroinflammatory diseases connected to dysfunction in the gut [68]. As mentioned earlier, an increased amount of glucose in the blood may lead to T2DM. Researchers are trying to link and understand the connection between conditions like hyperinsulinemia and T2DM and the threshold of GLP-1 with neuroinflammation and neurodegeneration (Figure 2).

With progress in this area, researchers will be able to discover whether GLP-1 and its agonists act as barriers or protectants against neuroinflammatory diseases, as it has been found that higher levels of GLP-1 are neuroprotective, as well as improve the functional outcomes of cells [68]. GLP-1 receptor (GLP-1R) agonists can regulate the microglia phenotype towards the M2 sub-type and, therefore, mitigate nerve cell apoptosis and inflammation-related damage [52]. GLP-1 has also been shown to have an anti-inflammatory effect on microglia, particularly by suppressing the M1 phenotype [72], which is linked with a pro-inflammatory response. Due to its ability to modulate microglial activation, GLP-1 and its receptor agonists should be investigated as potential treatments for inflammatory and neurodegenerative diseases. Researchers were also able to find that GLP-1, along with GIP, can cross the blood–brain barrier and bind to respective receptors in the CNS, where they have been shown to play significant roles regarding inflammation [52,53,73]. GIP agonists have been found to alleviate symptoms that are a result of neurodegenerative diseases that exhibit significant insulin resistance, like AD, by reducing the amount of neuronal plaque caused by neuroinflammation [52]. Accumulative studies also suggest that the homeostasis of metabolic peptides can influence energy metabolism and delay the development of neurodegenerative diseases, such as AD and PD, through anti-inflammatory, antioxidative, and antiapoptotic effects [74]. However, the dysregulation of hormonal peptides affects energy metabolism-related diseases, and people who are suffering from these disorders are reported to be at a higher risk of developing PD.

Studies performed on the metabolic peptide, PP, regarding neuroinflammation have allowed researchers to find a connection between the release of PP and the brain, as its receptors have been discovered in the vagus nerve [72,75,76,77]. It is important to note that when PP was directly injected into the dorsal motor nucleus of the vagus (DMV), postsynaptic connections were significantly reduced. It was also discovered that PP acts on postganglionic cholinergic neurons to prevent acetylcholine release [78]. Knowing this, investigators can begin to assume that an excessive amount of PP can eventually lead to neurodegeneration or the development of a neuroinflammatory disease (Figure 2). Studies have shown that PP could be significantly decreased in individuals with PD, potentially indicating a connection between the pancreas and the neurological symptoms of the disease, particularly related to vagus nerve function and the gastrointestinal issues often experienced by PD patients [79,80]. Progress made on these topics regarding metabolic peptides and neuroinflammation will be extremely important, as there have been groundbreaking discoveries about connections between conditions like diabetes and the crucial peptides involved in its diagnosis and neuroinflammatory and neurodegenerative diseases.

## 7. Metabolic Peptides in T3cDM and Dementia

Type 3 diabetes has been interpreted by some researchers to describe the concept that impaired insulin and insulin-like growth factor (IGF) signaling in the brain, similar to what happens in type 2 diabetes, may play a significant role in the development of AD [81,82]. Impairments in cerebral glucose utilization and energy metabolism represent very early abnormalities that accompany the initial stages of cognitive impairment [81]. This finding suggests a link between impaired insulin signaling and the pathogenesis of AD, raising the hypothesis that AD is related to type 3 diabetes. This hypothesis was tested by examining postmortem cases of advanced AD and detecting abnormal expressions of genes encoding insulin, as well as IGF peptides and their receptors [83], that correlated with the development and progression of lesions and dementia.

AD is one neuroinflammatory disease that has been frequently linked with T3cDM due to its significant insulin-resistant properties, as the disease causes a deficiency in insulin production at the synapses and signaling pathways needed for learning and memory [84,85]. It has been discovered that high concentrations of advanced glycation end products (AGEs), which develop plaques and neurofibrillary tangles, are found in both patients with higher hyperglycemic levels (T3cDM) and patients that have AD [86,87,88,89]. A variant of the so-called Alzheimer’s gene, *APOE4*, is also reported to interact with neuronal cells to use insulin, which may eventually cause the cells to starve and die. This phenomenon has been associated with T3cDM and more than 50% of AD cases could be linked to APOE4. Some researchers and scientists have begun to recognize AD as “diabetes of the brain”, or T3cDM, as it has been discovered that decreasing amounts of insulin in significant neuropathways causes detrimental effects on cognitive performance and the prevention of synaptic losses [90,91,92,93,94]. Research on the links between T3cDM and neuroinflammatory diseases like AD could become clearer and more comprehensible when connected with the research that is being undertaken on the link between GLP-1 and its neuroprotective and regenerative processes, regarding CNS damage and neuroinflammation [87,95,96].

GLP-1, GIP, and PP hormonal peptides are secreted in the gastrointestinal tract, where they influence insulin secretion. GLP-1 and GIP are thought to stimulate insulin release, while PP may have a more complex role depending on the glucose level and other unknown factors. On the other hand, GLP-1 tends to suppress glucagon secretion while GIP may stimulate it, depending on the microenvironment. Overall, the effects of these hormones are highly dependent on blood glucose level; under high-glucose conditions, GLP-1 and GIP primarily stimulate insulin secretion, and PP plays various roles depending on the metabolic state and surrounding environment. Patients that have dysfunction in the production and release of metabolic peptides like GLP-1, GIP, and PP are at a substantial risk of developing T3cDM and/or potentially developing a neurodegenerative disease like AD [97,98,99]. As stated previously, ineffective or low amounts of GLP-1 and extremely high levels of GIP cause the function of β cells to decrease, while also causing insulin resistance to increase (Figure 1). This activity can be linked to the neuroinflammation seen in patients with dementia, as studies have discovered that an increase in insulin concentration in the brain affects the neurotransmitters responsible for regulating long-term memory enhancement and long-term memory suppression, which is a common deficiency seen in AD resulting in impaired memory and cognition [97,98]. It has also been revealed that increased insulin resistance creates oxidative stress, and this can be linked back the development of the AGEs that form plaques.

Regarding the significant increase in the metabolic peptide PP, the resulting low amounts of insulin secretion can cause an accumulation of β-amyloid, increasing the chances of plaque formation and neurofibrillary tangles [98,100]. Researchers have been able to determine that insulin resistance can be linked to a decrease in glucose metabolism and glucose transporters in the brain, which are crucial for cognition and memory. They have also been able to discover that the dysfunction in the metabolic peptides responsible for insulin production and blood glucose regulation leads patients to have decreased levels of acetylcholine (ACh), which has been known to be associated with AD, as the plaques developed by the disease damage and break down cells that produce and use ACh. Research on the links between T3cDM and neuroinflammatory diseases like AD will become clearer and more comprehensible when connected with the research that is being undertaken on the link between GLP-1 and its neuroprotective and regenerative processes in relation to CNS damage and neuroinflammation [76,95].

## 8. Current Clinical Applications and Possible Future Research Directions

Findings from research performed in the past, as well as findings discovered more recently on these metabolic peptides, have allowed for the progression of more innovative clinical applications to transpire. The use of GLP-1-based treatment has expanded within recent years and has provided significant support to reducing the effects of not only obesity and diabetes, but also diseases that affect the cardiovascular system, renal function, liver function, and even sleep (specifically obstructive sleep apnea) [70,101,102]. Researchers have been able to more frequently advance GLP-1-focused therapies/treatments to manage specific conditions using GLP-1 and PP agonists, improving metabolic dysfunction with treatments like semaglutide, dulaglutide, liraglutide, and more. A simplified narrative delineating GLP-1, GIP, and PP hormonal peptides is presented in Table 1.

The incretin effects of GIP have sparked new development in current treatments for obesity, and by using GIP agonists, therapies have been developed to regulate glucose metabolism, increase β-cell function, and even manage/reduce systemic blood pressure [3,51]. For future research focuses, it has been recommended that GLP-1 and GIP treatments be combined with other receptor agonists or antagonists—preferably nutrient-based ones to allow for the most effective treatment to take place. The current treatment available, tirzepatide, is the only FDA-approved dual GLP-1/GIP receptor agonist. New emerging research has also allowed for the potential development of treatments regarding T3cDM and an understanding of its correlation to dementia. Metabolic peptides work together by binding to specific receptors on pancreatic β cells to manage/reduce insulin secretion and signaling, which ultimately reduces inflammation—specifically inflammation in the brain and within its processes—to help promote the survival of various brain cells [41,103]. With new clinical applications being developed so frequently regarding this topic, there seems to be endless opportunities for the development of future metabolic therapies.

## 9. Conclusions

Metabolic peptides have become a novel research area for the potential treatment of metabolic diseases such as obesity, diabetes, and hyperlipidemia. Obesity has affected children and adults all over the world and has continued to become more common each year. It is a disease that has been proven to develop into other life-altering conditions, such as hyperinsulinemia and T2DM, and researchers have been able to link conditions like T2DM to inflammatory diseases, specifically neuroinflammatory diseases. Some neuroinflammatory diseases result in significant abnormal amounts of metabolic peptides like GLP-1, GIP, and PP, which are crucial hormonal peptides needed to regulate insulin secretion, blood glucose level regulation, and gastric emptying; these processes are significantly dysregulated in patients with T2DM. Researchers have been able to link the dysfunction of these metabolic peptides to the connections between T2DM, PD, and AD. They have also presented many studies that have allowed them to see that the creation of GLP-1 agonists not only improves insulin production and encourages weight loss, but may also work as a neuroprotectant against neuroinflammation and neurodegeneration. There is still a lot of crucial research that must be undertaken, but there has been great progress regarding further understanding the links between metabolic peptides like GLP-1, GIP, and PP, as well as their agonists and properties, in order to combat obesity and neuroinflammation.

## Figures and Tables

**Table 1:** A summary table for the interactive functions of metabolic peptides GLP-1, GIP, and PP.

Functions	GLP-1	GIP	PP	References
Cells	Ineffective GLP-1 causes β cell function to decrease	Increased levels of GIP causes β cell function to decrease	Increased levels of PP causes F cell function to increase	[32,42,52,57]
	GLP-1 agonists allow pancreatic β cell apoptosis to decrease	GIP stimulates release of GLP-1 from α cells	β-cell marker is limited by expression in islet PP cells	
Insulin	Ineffective GLP-1 causes insulin resistance to increase	Increased levels of GIP causes insulin resistance to increase	Increased levels of PP causes insulin secretion to decrease	[17,41,42,47]
	GLP-1 agonists allow for insulin production to increase	Elevated GIP induces insulin resistance and promotes hypothalamic inflammation	Inhibits pancreatic exocrine secretion and acts as a brake on pancreatic secretion	
Obesity	GLP-1 suppresses glucagon production and delays gastric emptying, decreasing food intake	Excessive fat accumulation allows for the hypersecretion of GIP; may promote obesity	PP regulates metabolism and delays gastric emptying, decreasing food intake	[38,48,57]
Type 2 Diabetes	Ineffective GLP-1 causes individuals to have significantly lower glycemic control responses	There is high resistance to the effect of GIP in the bodies of patients with Type 2 Diabetes	High levels of PP are secreted from the pancreas in patients with Type 2 Diabetes	[39,40,47,57]
	GLP-1 agonists lower glucose levels and manage	GIP acts as a protection against hypoglycemia	PP may act as a counter-regulatory mechanism to help	

## References

[1] Collins L., Costello R.A. (2024). Glucagon-Like Peptide-1 Receptor Agonists. StatPearls.

[2] Meloni A.R., DeYoung M.B., Lowe C., Parkes D.G. (2013). GLP-1 receptor activated insulin secretion from pancreatic β-cells: Mechanism and glucose dependence. Diabetes Obes. Metab..

[3] Zheng Z., Zong Y., Ma Y., Tian Y., Pang Y., Zhang C., Gao J. (2024). Glucagon-like peptide-1 receptor: Mechanisms and advances in therapy. Signal Transduct. Target. Ther..

[4] Carlessi R., Chen Y., Rowlands J., Cruzat V.F., Keane K.N., Egan L., Mamotte C., Stokes R., Gunton J.E., Bittencourt P.I.H. (2017). GLP-1 receptor signalling promotes β-cell glucose metabolism via mTOR-dependent HIF-1α activation. Sci. Rep..

[5] Rowlands J., Heng J., Newsholme P., Carlessi R. (2018). Pleiotropic Effects of GLP-1 and Analogs on Cell Signaling, Metabolism, and Function. Front. Endocrinol..

[6] Kuhre R.E., Deacon C.F., Holst J.J., Petersen N. (2021). What Is an L-Cell and How Do We Study the Secretory Mechanisms of the L-Cell?. Front. Endocrinol..

[7] Cabou C., Burcelin R. (2011). GLP-1, the gut-brain, and brain-periphery axes. Rev. Diabet. Stud..

[8] Wachsmuth H.R., Weninger S.N., Duca F.A. (2022). Role of the gut-brain axis in energy and glucose metabolism. Exp. Mol. Med..

[9] Sharma D., Verma S., Vaidya S., Kalia K., Tiwari V. (2018). Recent updates on GLP-1 agonists: Current advancements & challenges. Biomed. Pharmacother..

[10] Mariam Z., Niazi S.K. (2024). Glucagon-like peptide agonists: A prospective review. Endocrinol. Diabetes Metab..

[11] Gupta K., Raja A. (2024). Physiology, Gastric Inhibitory Peptide. StatPearls.

[12] McIntosh C.H., Widenmaier S., Kim S.J. (2009). Glucose-dependent insulinotropic polypeptide (Gastric Inhibitory Polypeptide; GIP). Vitam. Horm..

[13] Nauck M.A., Meier J.J. (2018). Incretin hormones: Their role in health and disease. Diabetes Obes. Metab..

[14] Kagdi S., Lyons S.A., Beaudry J.L. (2024). The interplay of glucose-dependent insulinotropic polypeptide in adipose tissue. J. Endocrinol..

[15] Seino Y., Fukushima M., Yabe D. (2010). GIP and GLP-1, the two incretin hormones: Similarities and differences. J. Diabetes Investig..

[16] Samms R.J., Coghlan M.P., Sloop K.W. (2020). How May GIP Enhance the Therapeutic Efficacy of GLP-1?. Trends Endocrinol. Metab..

[17] Zhu W., Tanday N., Flatt P.R., Irwin N. (2023). Pancreatic polypeptide revisited: Potential therapeutic effects in obesity-diabetes. Peptides.

[18] Cuenco J., Minnion J., Tan T., Scott R., Germain N., Ling Y., Chen R., Ghatei M., Bloom S. (2017). Degradation Paradigm of the Gut Hormone, Pancreatic Polypeptide, by Hepatic and Renal Peptidases. Endocrinology.

[19] Goyal R.K., Guo Y., Mashimo H. (2019). Advances in the physiology of gastric emptying. Neurogastroenterol. Motil..

[20] (2008). Proceedings from the 2008 Meeting of the Society for the Study of Ingestive Behavior—SSIB 2008, Paris, France, 15–19 July 2008.

[21] Fitch A.K., Bays H.E. (2022). Obesity definition, diagnosis, bias, standard operating procedures (SOPs), and telehealth: An Obesity Medicine Association (OMA) Clinical Practice Statement (CPS) 2022. Obes. Pillars.

[22] Lin X., Li H. (2021). Obesity: Epidemiology, Pathophysiology, and Therapeutics. Front. Endocrinol..

[23] Safaei M., Sundararajan E.A., Driss M., Boulila W., Shapi’i A. (2021). A systematic literature review on obesity: Understanding the causes & consequences of obesity and reviewing various machine learning approaches used to predict obesity. Comput. Biol. Med..

[24] Balasundaram P., Krishna S. (2024). Obesity Effects on Child Health. StatPearls.

[25] Jebeile H., Kelly A.S., O’Malley G., Baur L.A. (2022). Obesity in children and adolescents: Epidemiology, causes, assessment, and management. Lancet Diabetes Endocrinol..

[26] Masood B., Moorthy M. (2023). Causes of obesity: A review. Clin. Med..

[27] Poulain M., Doucet M., Major G.C., Drapeau V., Series F., Boulet L.P., Tremblay A., Maltais F. (2006). The effect of obesity on chronic respiratory diseases: Pathophysiology and therapeutic strategies. CMAJ.

[28] Kivimaki M., Strandberg T., Pentti J., Nyberg S.T., Frank P., Jokela M., Ervasti J., Suominen S.B., Vahtera J., Sipila P.N. (2022). Body-mass index and risk of obesity-related complex multimorbidity: An observational multicohort study. Lancet Diabetes Endocrinol..

[29] Singh D.D., Shati A.A., Alfaifi M.Y., Elbehairi S.E.I., Han I., Choi E.H., Yadav D.K. (2022). Development of Dementia in Type 2 Diabetes Patients: Mechanisms of Insulin Resistance and Antidiabetic Drug Development. Cells.

[30] Klein S., Gastaldelli A., Yki-Jarvinen H., Scherer P.E. (2022). Why does obesity cause diabetes?. Cell Metab..

[31] Murotani T., Ishizuka T., Isogawa Y., Karashima M., Yamatodani A. (2011). Possible involvement of serotonin 5-HT2 receptor in the regulation of feeding behavior through the histaminergic system. Neuropharmacology.

[32] Galicia-Garcia U., Benito-Vicente A., Jebari S., Larrea-Sebal A., Siddiqi H., Uribe K.B., Ostolaza H., Martin C. (2020). Pathophysiology of Type 2 Diabetes Mellitus. Int. J. Mol. Sci..

[33] Reed J., Bain S., Kanamarlapudi V. (2021). A Review of Current Trends with Type 2 Diabetes Epidemiology, Aetiology, Pathogenesis, Treatments and Future Perspectives. Diabetes Metab. Syndr. Obes..

[34] Thomas D.D., Corkey B.E., Istfan N.W., Apovian C.M. (2019). Hyperinsulinemia: An Early Indicator of Metabolic Dysfunction. J. Endocr. Soc..

[35] Janssen J. (2021). Hyperinsulinemia and Its Pivotal Role in Aging, Obesity, Type 2 Diabetes, Cardiovascular Disease and Cancer. Int. J. Mol. Sci..

[36] Zhang A.M.Y., Wellberg E.A., Kopp J.L., Johnson J.D. (2021). Hyperinsulinemia in Obesity, Inflammation, and Cancer. Diabetes Metab. J..

[37] Zhang Q., Jin K., Chen B., Liu R., Cheng S., Zhang Y., Lu J. (2022). Overnutrition Induced Cognitive Impairment: Insulin Resistance, Gut-Brain Axis, and Neuroinflammation. Front. Neurosci..

[38] Andersen A., Lund A., Knop F.K., Vilsboll T. (2018). Glucagon-like peptide 1 in health and disease. Nat. Rev. Endocrinol..

[39] Calanna S., Christensen M., Holst J.J., Laferrere B., Gluud L.L., Vilsboll T., Knop F.K. (2013). Secretion of glucagon-like peptide-1 in patients with type 2 diabetes mellitus: Systematic review and meta-analyses of clinical studies. Diabetologia.

[40] Karakasis P., Patoulias D., Tzeis S., Fragakis N. (2024). Glucagon-Like Peptide-1 Receptor Agonists and Atrial Fibrillation Recurrence After Ablation: A Fire Without the Smoke?. JACC Clin. Electrophysiol..

[41] Peterson S.M., Juliana C.A., Hu C.F., Chai J., Holliday C., Chan K.Y., Lujan Hernandez A.G., Challocombe Z., Wang L., Han Z. (2023). Optimization of a Glucagon-Like Peptide 1 Receptor Antagonist Antibody for Treatment of Hyperinsulinism. Diabetes.

[42] Rorsman P., Ashcroft F.M. (2018). Pancreatic β-Cell Electrical Activity and Insulin Secretion: Of Mice and Men. Physiol. Rev..

[43] Zhang D., Lv G. (2018). Therapeutic potential of spinal GLP-1 receptor signaling. Peptides.

[44] Figlioli G., Piovani D., Peppas S., Pugliese N., Hassan C., Repici A., Lleo A., Aghemo A., Bonovas S. (2024). Glucagon-Like Peptide-1 Receptor Agonists and Risk of Gastrointestinal Cancers: A Systematic Review and Meta-Analysis of Randomized Controlled Trials. Pharmacol. Res..

[45] Irwin N., Flatt P.R. (2009). Therapeutic potential for GIP receptor agonists and antagonists. Best Pract. Res. Clin. Endocrinol. Metab..

[46] Holst J.J., Rosenkilde M.M. (2020). GIP as a Therapeutic Target in Diabetes and Obesity: Insight From Incretin Co-Agonists. J. Clin. Endocrinol. Metab..

[47] Miyawaki K., Yamada Y., Ban N., Ihara Y., Tsukiyama K., Zhou H., Fujimoto S., Oku A., Tsuda K., Toyokuni S. (2002). Inhibition of gastric inhibitory polypeptide signaling prevents obesity. Nat. Med..

[48] Zandvakili I., Perez-Tilve D. (2024). The unexpected role of GIP in transforming obesity treatment. Trends Endocrinol. Metab..

[49] Ciardullo S., Morieri M.L., Daniele G., Fiorentino T.V., Mezza T., Trico D., Consoli A., Del Prato S., Giorgino F., Piro S. (2024). GLP1-GIP receptor co-agonists: A promising evolution in the treatment of type 2 diabetes. Acta Diabetol..

[50] Spielman L.J., Little J.P., Klegeris A. (2014). Inflammation and insulin/IGF-1 resistance as the possible link between obesity and neurodegeneration. J. Neuroimmunol..

[51] Liu Q.K. (2024). Mechanisms of action and therapeutic applications of GLP-1 and dual GIP/GLP-1 receptor agonists. Front. Endocrinol..

[52] Kopp K.O., Glotfelty E.J., Li Y., Greig N.H. (2022). Glucagon-like peptide-1 (GLP-1) receptor agonists and neuroinflammation: Implications for neurodegenerative disease treatment. Pharmacol. Res..

[53] Nowell J., Blunt E., Edison P. (2023). Incretin and insulin signaling as novel therapeutic targets for Alzheimer’s and Parkinson’s disease. Mol. Psychiatry.

[54] Louie D.S., Williams J.A., Owyang C. (1985). Action of pancreatic polypeptide on rat pancreatic secretion: In vivo and in vitro. Am. J. Physiol..

[55] Lonovics J., Devitt P., Watson L.C., Rayford P.L., Thompson J.C. (1981). Pancreatic polypeptide. A review. Arch. Surg..

[56] Tiscornia O.M., Negri G.A., Otero G., Lopez Mingorance F.N., Waisman H., Tiscornia-Wasserman P.G. (2015). Pancreatic polypeptide: A review of its involvement in neuro-endocrine reflexes, islet-acinar interactions and ethanol-evoked physiopatologic pancreatic gland changes. Acta Gastroenterol. Latinoam..

[57] Nagpal S.J.S., Bamlet W.R., Kudva Y.C., Chari S.T. (2018). Comparison of Fasting Human Pancreatic Polypeptide Levels Among Patients With Pancreatic Ductal Adenocarcinoma, Chronic Pancreatitis, and Type 2 Diabetes Mellitus. Pancreas.

[58] Aslam M., Vijayasarathy K., Talukdar R., Sasikala M., Nageshwar Reddy D. (2020). Reduced pancreatic polypeptide response is associated with early alteration of glycemic control in chronic pancreatitis. Diabetes Res. Clin. Pract..

[59] Brunicardi F.C., Chaiken R.L., Ryan A.S., Seymour N.E., Hoffmann J.A., Lebovitz H.E., Chance R.E., Gingerich R.L., Andersen D.K., Elahi D. (1996). Pancreatic polypeptide administration improves abnormal glucose metabolism in patients with chronic pancreatitis. J. Clin. Endocrinol. Metab..

[60] Ramsey M.L., Conwell D.L., Hart P.A. (2017). Complications of Chronic Pancreatitis. Dig. Dis. Sci..

[61] Nair R.J., Lawler L., Miller M.R. (2007). Chronic pancreatitis. Am. Fam. Physician.

[62] Rabiee A., Galiatsatos P., Salas-Carrillo R., Thompson M.J., Andersen D.K., Elahi D. (2011). Pancreatic polypeptide administration enhances insulin sensitivity and reduces the insulin requirement of patients on insulin pump therapy. J. Diabetes Sci. Technol..

[63] Bandala C., Cardenas-Rodriguez N., Reyes-Long S., Cortes-Altamirano J.L., Garciadiego-Cazares D., Lara-Padilla E., Ibanez-Cervantes G., Mancilla-Ramirez J., Gomez-Manzo S., Alfaro-Rodriguez A. (2022). Trends in Gliosis in Obesity, and the Role of Antioxidants as a Therapeutic Alternative. Antioxidants.

[64] Bolon B. (2023). Toxicologic Pathology Forum Opinion: Interpretation of Gliosis in the Brain and Spinal Cord Observed During Nonclinical Safety Studies. Toxicol. Pathol..

[65] Amlerova Z., Chmelova M., Anderova M., Vargova L. (2024). Reactive gliosis in traumatic brain injury: A comprehensive review. Front. Cell. Neurosci..

[66] Papadimitriou D., Le Verche V., Jacquier A., Ikiz B., Przedborski S., Re D.B. (2010). Inflammation in ALS and SMA: Sorting out the good from the evil. Neurobiol. Dis..

[67] Silver J., Miller J.H. (2004). Regeneration beyond the glial scar. Nat. Rev. Neurosci..

[68] Polcyn R., Capone M., Matzelle D., Hossain A., Chandran R., Banik N.L., Haque A. (2020). Enolase inhibition alters metabolic hormones and inflammatory factors to promote neuroprotection in spinal cord injury. Neurochem. Int..

[69] Holscher C. (2014). The incretin hormones glucagonlike peptide 1 and glucose-dependent insulinotropic polypeptide are neuroprotective in mouse models of Alzheimer’s disease. Alzheimer’s Dement..

[70] Campbell J.E., Drucker D.J. (2013). Pharmacology, physiology, and mechanisms of incretin hormone action. Cell Metab..

[71] Pekny M., Pekna M. (2016). Reactive gliosis in the pathogenesis of CNS diseases. Biochim. Biophys. Acta (BBA) Mol. Basis Dis..

[72] Diz-Chaves Y., Mastoor Z., Spuch C., Gonzalez-Matias L.C., Mallo F. (2022). Anti-Inflammatory Effects of GLP-1 Receptor Activation in the Brain in Neurodegenerative Diseases. Int. J. Mol. Sci..

[73] Holscher C. (2020). Brain insulin resistance: Role in neurodegenerative disease and potential for targeting. Expert Opin. Investig. Drugs.

[74] Liu M., Jiao Q., Du X., Bi M., Chen X., Jiang H. (2021). Potential Crosstalk Between Parkinson’s Disease and Energy Metabolism. Aging Dis..

[75] Yoon G., Kim Y.K., Song J. (2020). Glucagon-like peptide-1 suppresses neuroinflammation and improves neural structure. Pharmacol. Res..

[76] Chen B., Yu X., Horvath-Diano C., Ortuno M.J., Tschop M.H., Jastreboff A.M., Schneeberger M. (2024). GLP-1 programs the neurovascular landscape. Cell Metab..

[77] Zhang W., Xiao D., Mao Q., Xia H. (2023). Role of neuroinflammation in neurodegeneration development. Signal Transduct. Target. Ther..

[78] Phillips P.A., Yang L., Shulkes A., Vonlaufen A., Poljak A., Bustamante S., Warren A., Xu Z., Guilhaus M., Pirola R. (2010). Pancreatic stellate cells produce acetylcholine and may play a role in pancreatic exocrine secretion. Proc. Natl. Acad. Sci. USA.

[79] Knudsen K., Hartmann B., Fedorova T.D., Ostergaard K., Krogh K., Moller N., Holst J.J., Borghammer P. (2017). Pancreatic Polypeptide in Parkinson’s Disease: A Potential Marker of Parasympathetic Denervation. J. Parkinson’s Dis..

[80] Zheng Y., Zhang L., Xie J., Shi L. (2021). The Emerging Role of Neuropeptides in Parkinson’s Disease. Front. Aging Neurosci..

[81] Sims N.R., Bowen D.M., Smith C.C., Flack R.H., Davison A.N., Snowden J.S., Neary D. (1980). Glucose metabolism and acetylcholine synthesis in relation to neuronal activity in Alzheimer’s disease. Lancet.

[82] de la Monte S.M., Wands J.R. (2008). Alzheimer’s disease is type 3 diabetes-evidence reviewed. J. Diabetes Sci. Technol..

[83] Steen E., Terry B.M., Rivera E.J., Cannon J.L., Neely T.R., Tavares R., Xu X.J., Wands J.R., de la Monte S.M. (2005). Impaired insulin and insulin-like growth factor expression and signaling mechanisms in Alzheimer’s disease--is this type 3 diabetes?. J. Alzheimer’s Dis..

[84] Viola K.L., Velasco P.T., Klein W.L. (2008). Why Alzheimer’s is a disease of memory: The attack on synapses by Aβ oligomers (ADDLs). J. Nutr. Health Aging.

[85] Kroner Z. (2009). The relationship between Alzheimer’s disease and diabetes: Type 3 diabetes?. Altern. Med. Rev..

[86] Yamagishi S., Ueda S., Okuda S. (2007). Food-derived advanced glycation end products (AGEs): A novel therapeutic target for various disorders. Curr. Pharm. Des..

[87] Nguyen T.T., Ta Q.T.H., Nguyen T.K.O., Nguyen T.T.D., Giau V.V. (2020). Type 3 Diabetes and Its Role Implications in Alzheimer’s Disease. Int. J. Mol. Sci..

[88] Takeuchi M., Yamagishi S. (2008). Possible involvement of advanced glycation end-products (AGEs) in the pathogenesis of Alzheimer’s disease. Curr. Pharm. Des..

[89] Zhu X., Su B., Wang X., Smith M.A., Perry G. (2007). Causes of oxidative stress in Alzheimer disease. Cell. Mol. Life Sci..

[90] Li J., Bai L., Wei F., Zhao J., Wang D., Xiao Y., Yan W., Wei J. (2019). Therapeutic Mechanisms of Herbal Medicines Against Insulin Resistance: A Review. Front. Pharmacol..

[91] Matioli M., Nitrini R. (2015). Mechanisms linking brain insulin resistance to Alzheimer’s disease. Dement. Neuropsychol..

[92] McAteer M.A., Choudhury R.P. (2009). Chapter 4—Applications of nanotechnology in molecular imaging of the brain. Prog. Brain Res..

[93] Lillioja S., Mott D.M., Spraul M., Ferraro R., Foley J.E., Ravussin E., Knowler W.C., Bennett P.H., Bogardus C. (1993). Insulin resistance and insulin secretory dysfunction as precursors of non-insulin-dependent diabetes mellitus. Prospective studies of Pima Indians. N. Engl. J. Med..

[94] Razay G., Vreugdenhil A., Wilcock G. (2006). Obesity, abdominal obesity and Alzheimer disease. Dement. Geriatr. Cogn. Disord..

[95] Eakin K.A., Saleem M., Herrmann N., Cogo-Moreira H., Mielke M.M., Oh P.I., Haughey N.J., Venkata S.L.V., Lanctot K.L., Swardfager W. (2019). Plasma Sphingolipids Mediate a Relationship Between Type 2 Diabetes and Memory Outcomes in Patients with Coronary Artery Disease Undertaking Exercise. J. Alzheimer’s Dis..

[96] Mittal K., Mani R.J., Katare D.P. (2016). Type 3 Diabetes: Cross Talk between Differentially Regulated Proteins of Type 2 Diabetes Mellitus and Alzheimer’s Disease. Sci. Rep..

[97] Kandimalla R., Thirumala V., Reddy P.H. (2017). Is Alzheimer’s disease a Type 3 Diabetes? A critical appraisal. Biochim. Biophys. Acta (BBA) Mol. Basis Dis..

[98] Michailidis M., Moraitou D., Tata D.A., Kalinderi K., Papamitsou T., Papaliagkas V. (2022). Alzheimer’s Disease as Type 3 Diabetes: Common Pathophysiological Mechanisms between Alzheimer’s Disease and Type 2 Diabetes. Int. J. Mol. Sci..

[99] Nisar O., Pervez H., Mandalia B., Waqas M., Sra H.K. (2020). Type 3 Diabetes Mellitus: A Link Between Alzheimer’s Disease and Type 2 Diabetes Mellitus. Cureus.

[100] Ahn H.J., Zamolodchikov D., Cortes-Canteli M., Norris E.H., Glickman J.F., Strickland S. (2010). Alzheimer’s disease peptide β-amyloid interacts with fibrinogen and induces its oligomerization. Proc. Natl. Acad. Sci. USA.

[101] Drucker D.J. (2024). Expanding applications of therapies based on GLP1. Nat. Rev. Endocrinol..

[102] Muller T.D., Finan B., Bloom S.R., D’Alessio D., Drucker D.J., Flatt P.R., Fritsche A., Gribble F., Grill H.J., Habener J.F. (2019). Glucagon-like peptide 1 (GLP-1). Mol. Metab..

[103] Yu C.J., Ma D., Song L.L., Zhai Z.N., Tao Y., Zhang Y., Cai L.Y., Hou Y.H., Chen H.Y., Wang L. (2020). The role of GLP-1/GIP receptor agonists in Alzheimer’s disease. Adv. Clin. Exp. Med..

